# Development of an Innovative Pipeline With Fusion, Digital Planning, and Three‐Dimensional Printing to Improve Mitral Valve Interventional Care

**DOI:** 10.1111/echo.70184

**Published:** 2025-05-20

**Authors:** Jelle P. Man, Berto J. Bouma, Folkert Asselbergs, Steven A. J. Chamuleau, Mark J. Schuuring

**Affiliations:** ^1^ Department of Cardiology Amsterdam UMC, Heart Center Amsterdam the Netherlands; ^2^ Netherlands Heart Institute Amsterdam the Netherlands; ^3^ Institute of Health Informatics University College London London UK; ^4^ The National Institute for Health Research University College London Hospitals Biomedical Research Centre University College London London UK; ^5^ Department of Cardiology Medical Spectrum Twente Enschede the Netherlands; ^6^ Department of Biomedical Signals and Systems University of Twente Enschede the Netherlands

**Keywords:** 3D printing, fusion workflow, image registration, mitral valve repair, mitral valve, rapid prototyping

## Abstract

**Aim:**

Transesophageal echocardiography (TEE) is the modality of choice for mitral valve (MV) interventional planning. However, computed tomography (CT) has been proposed as a standard screening tool for MV interventions to assess the risk of injury to the left circumflex artery. We aimed to develop a pipeline to fuse CT and MV modalities and create three‐dimensional (3D) printable models of the MV apparatus to enhance interventional planning and assessed its usability among clinicians.

**Methods and Results:**

The design, production, and assessment of the 3D‐printed personalized models were based on TEE enriched with data from CT. The digital pipeline consisted of fusion with a mutual information algorithm using 3D Slicer (Slicer) and Elastix toolboxes. Flexible 3D printing was performed using Agilus 30 Clear. The pipeline was feasible for achieving semiautomatic fusion of anatomical structures related to MV interventional planning. Visualization of fused synergistic information for 3D planning could be provided in three distinct cases of MV regurgitation (secondary MR with tenting, flail leaflet, and prolapse). The 3D printing resulted in a flexibility in line with actual tissue allowing for tactile modification. An average System Usability Score of 71 indicates a moderately good usability among imaging cardiologists, intervention cardiologists, and cardiac surgeons.

**Conclusion:**

The presented pipeline with digital planning and personalized 3D printing has reached the technology readiness level for application in mitral valve interventions, and prospective clinical trials with endpoints of surgical effectiveness. Comprehensive and efficient interactions between clinicians and technical staff are essential to establish well‐designed patient‐specific interventions.

## Graphical abstract

The innovative pipeline to improve mitral valve interventional care. Panel A: separate visualizations. Panel B: visualizations of the feasibility test and a picture of the three‐dimensional (3D) printed patient‐specific model.

The present article describes a semiautomatic fusion pipeline for anatomical structures related to MV interventional planning. The study evaluated the feasibility of this pipeline and assessed its usability among clinicians.

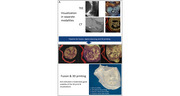

## 1 Introduction

Mitral valve regurgitation (MR) is the most common heart valve disease in developed nations and is associated with a substantially increased risk of mortality [1, 2, 3]. Mitral valve (MV) surgery is the recommended treatment by the European Society of Cardiology for patients with severe primary MR and secondary MR after optimal medical therapy. Three‐dimensional (3D) planning and printing in MV surgery (MVS) is a promising tool for operators to gain an enhanced intuition of the patient‐specific anatomy, especially in the training for novel (minimally invasive) surgical procedures and specialists in training [4]. As preparation for MV surgery, transesophageal echocardiography (TEE) is the modality of choice for surgical planning. Additionally, computed tomography (CT) was proposed as a standard modality prior to surgery to assess the location of the left circumflex artery (LCx) with respect to the MV [5]. Considering the usefulness of both modalities in imaging of the mitral valve apparatus, we hypothesized that an effective fusion of CT and TEE modalities could be useful to enhance surgical planning. Technical limitations to fusion pipelines and only recently developed innovations in flexible 3D printing have contributed to the limited implementation of patient‐specific models thus far. We present a pipeline to fuse CT and MV modalities and create 3D printable flexible models of the MV and relevant surrounding anatomy derived from both modalities and assessed its feasibility and usability among clinicians.

## 2 Methods

### 2.1 Data Acquisition for Fusion Pipeline

Patients with severe MR who were scheduled for an intervention were considered for participation in the feasibility analysis of the developed fusion pipeline. A single case has previously been published [6]. Eligible patients have received a TEE with 3D acquisition of the mitral valve and CG‐gated cardiac CT as standard workup for their MV interventions. Three patients were included in this retrospective analysis. Patients were selected based on the presence of three distinct pathophysiological (sub)types of MR. TEE assessments were obtained using GE systems (GE Vingmed Ultrasound, Horten, Norway); CT acquisitions were obtained using Siemens systems (Somatom Force, Munich, Germany). The 3D TEE acquisition is standardly stored in private DICOM fields not suitable for loading in non‐GE applications. In order to load exported 3D TEE DICOM files Image3dAPI (GE, Munich, Germany) was used in order to load the file in Slicer3D (Slicer). This retrospective study was approved by the local Human Research Ethics Committee, and informed consent was obtained.

### 2.2 Fusion Workflow

**FIGURE 1:**
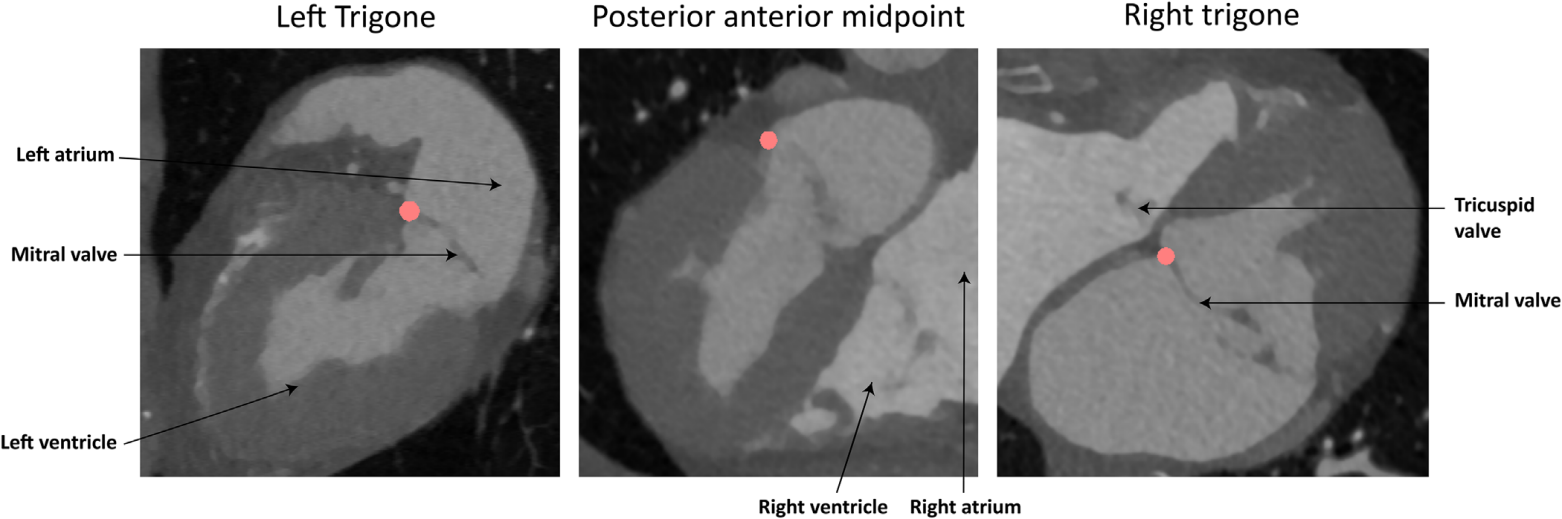
The CT slices display the location of the left trigone, posterior–anterior midpoint, and right trigone of the mitral valve.

**FIGURE 2:**
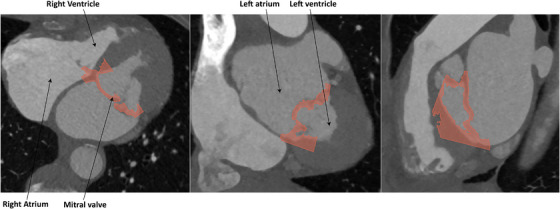
Fusion overlay after the semiautomatic algorithm. A TEE segmentation of the mitral valve is transparently visualized as an overlay over CT slices.

In Slicer3D, corresponding systolic timeframes were selected in CT and TEE acquisitions for all patients. The medial and lateral trigone and the posterior–anterior midpoint of the MV were identified and marked in both the CT and TEE acquisitions (Figure 1) [7, 8, 9]. The scans were prealigned using the selected landmarks as input for the Procrustes algorithm [10]. The CT scan is subsequently cropped to the size of the TEE, and a fusion of the TEE scan to the cropped CT scan is performed using a mutual algorithm with an adaptive gradient descent optimizer [11]. This algorithm is applied using Elastix software (Utrecht University Medical Center, Utrecht, the Netherlands) [12]. The parameter settings for this algorithm are provided in the supplementary materials. The quality of the fusion was checked by an imaging cardiologist and it was determined if manual corrections were needed (an overlay of the scans is provided in Figure 2). The left coronary artery and myocardium on CT and the mitral valve with surrounding anatomy on TEE are segmented on the aligned scans. This is done using the artificial intelligence model TotalSegmentator of Slicer with manual corrections if needed [13, 14].

### 2.3 Flexible 3D Printing

The reconstructions from both CT and TEE imaging were exported from Slicer3D to Surface Tessellation Language (STL) files. The reconstructions are merged to one combined reconstruction using the union operator of Meshmixer (Autodesk, Mill Valley, USA). This combined reconstruction is hollowed and smoothed using Meshmixer and the Poisson disk sampling tool of Meshlab (Istituto di Scienza e Tecnologie dell'Informazione, Pisa, Italy) [15, 16, 17]. To expose the LCx, an indent in the tissue surrounding the LCx was made, using extrusion along the normal vectors of the 3D model. At the apex of the heart and at the left lateral side a small window in the ventricle was created for visibility. This manual editing was done in Meshmixer. To achieve the desired flexibility, the 3D model was digitally hollowed with a wall thickness of 1.2 mm [16]. The model was made suitable for 3D printing using 3D builder (Microsoft, Redmond, USA). One clinical case is 3D printed, using flexible semitranslucent material with walls of 1.2 mm in thickness and printing with Agilus 30 Clear (with a shore A value of 30), to test the feasibility of 3D printing of fused scans. Materials were chosen based on prior research [16]. The steps required for fusion and 3D printing are shown in Table 1.

**TABLE 1:** Image fusion and 3D printing steps.

Image fusion		
Process step	Software used	Manual action
Initial alignment	Slicer (Procrustes)	Selecting 3 landmarks
Automatic alignment	Elastix (Mutual information algorithm)	
3D printing		
Segmenting reconstructions	Slicer	Manual editing of automatic segmentation
Automatic and manual smoothing	Meshlab and Meshmixer	
Editing model	Meshmixer	Make an indent at the location of the LCx and a hole through the atrium for visualization
Hollowing to create walls of 1.2 mm thickness	Meshmixer	

Abbreviations: 3D, three‐dimensional; LCx, left circumflex artery.

### 2.4 Questionnaires on Usability

Clinical assessment was performed with System Usability Score (SUS) questionnaires among 14 clinicians involved in multidisciplinary decision making for MV interventions. None of the respondents were involved in the development of the fusion pipeline or the research article. The SUS has been validated as a tool to test the usability and it is proven to be robust to slight adaptations to apply it to various applications [18]. It is therefore chosen as the methodology of choice to evaluate the usability of these patient‐specific 3D digital visualizations and 3D prints. Scores are calculated by the following formula:

- Positively formulated questions: Score = (answer–1) 2.5
- Negatively formulated questions: Score = 5–(answer–1) 2.5

Data was reported using mean values and standard deviations or median and interquartile range if appropriate. A separate analysis was conducted for mitral valve surgeons. All respondents were involved in Heart Team evaluations related to mitral valve interventions and were actively treating patients with valvular heart diseases. Respondents are contacted by means of individual and group meetings with mitral valve specialists from three Dutch centers (Amsterdam University Medical Center, Amsterdam, the Netherlands; Medical Spectrum Twente, Enschede, the Netherlands; University Medical Center Groningen, Groningen, the Netherlands). Other feedback was acquired during the 2023 European Association of Cardiovascular Imaging (EACVI) conference. Other than the SUS questionnaire, respondents were asked to provide the following information: institution, sex, years of experience in mitral valve interventional care (separated in overall experience and in the interpretation of TEE and CT images), number of patients treated in the last 3 months, number of performed mitral valve interventions. Respondents also had the opportunity to leave free‐text input. Raised issues or feedback points were grouped and reported.

## 3 Results

The developed pipeline was effective in achieving semiautomatic fusion of CT and TEE visualizations of the anatomy relevant to MV interventions. Visualization of fused synergistic information for 3D planning could be provided in three distinct cases of MV regurgitation (secondary MR with tenting, flail leaflet, and prolapse; Graphical abstract, panel B). The authors did not encounter procedural problems with the application of this pipeline in this feasibility test. The applied mutual information algorithm required no manual adjustments, assessed by trained medical imagers and cardiologists.

The 3D printing resulted in a flexibility comparable to tissue allowing for tactile modification for interventional planning in line with the results of prior research. The printing process required some manual editing of the 3D model to improve visualization of the LCx, smoothing of the model to make it suitable for 3D printing, and hollowing of the 3D model to achieve the desired flexibility. During this manual editing process, no complications or difficulties were encountered. The methodology resulted in a clear visualization of the LCx with respect to the 3D‐printed MV.

The SUS was 71/100 arbitrary units (AU), indicating a moderately good usability of the 3D print and visualizations. The separate analysis in surgeons resulted in a SUS of 72/100 AU and thus a similar SUS. Scores on the individual questionnaires and additional information about the respondents are summarized in Tables 2 and 3. In the free text input, respondents indicated the following points:

- The flexibility of the model is of added value to potentially test surgical or interventional techniques.
- Respondents pointed out the added value of the visualized and 3D‐printed papillary muscles.
- Respondents expressed the usability of these 3D prints for training purposes of novel interventional treatments.
- Respondents indicated the need for manual separation of the valvular leaflets based on temporal information on the TEE to improve the model.
- Some respondents felt that the 3D print was only warranted in a limited number of cases. Or for the training of inexperienced surgeons or novel surgical techniques
- Clinicians suggested to include an indication of the directionality of the regurgitation in the 3D model.
- The suggestion was given to add color on the 3D print to mark pathological areas.

**TABLE 2:** SUS questionnaire.

	SUS (0–100)	
Question	mean	SD
I think that I would like to use 3D prints and 3D visualizations like this frequently.	61	±25
I find the 3D print and 3D visualizations unnecessarily complex.	75	±22
I thought the 3D print and 3D visualizations were easy to understand.	73	±18
I think that I would need the support of a technical person to be able to use this 3D print or 3D visualizations in practice.	73	±23
I found that the underlying anatomy was well integrated into the 3D models.	71	±30
I would imagine that most people would learn to use 3D prints and 3D visualizations like this quickly.	75	±24
I found the 3D print very cumbersome/unwieldy to use.	64	±29
I felt very confident using the 3D print and examining the 3D visualizations.	73	±21
I needed to learn a lot of things before I could get going with this 3D print and the 3D visualizations.	71	±31
Total SUS	**71**	**±16**
Total SUS among surgeons	**72**	**±20**

*Note*: These scores are the most important and therefore portrayed in bold.

Abbreviations: 3D, three‐dimensional; SUS, System Usability Score.

**TABLE 3:** Additional information about respondents.

Function	
surgeon	8 (57%)
Imaging cardiologist in MV intervention team	5 (36%)
Interventional cardiologist	1 (7%)
Experience in MV interventional care	
1–5 years	4 (29%)
5–10 years	4 (29%)
>10 years	6 (43%)
Number of performed interventions	
None	5 (36%)
10—30	1 (7%)
30—100	3 (21%)
>100	5 (36%)
Sex	
Male	11 (79%)
female	3 (21%)

Abbreviation: MV, mitral valve.

## 4 Discussion

In this article, a novel pipeline for 3D fusion and printing is presented, and its feasibility was tested. Visualizations of fused synergistic information for 3D planning could be provided in three distinct cases of MV regurgitation (secondary MR with tenting, flail leaflet, and prolapse). The applied mutual information algorithm required no manual adjustments, assessed by trained medical imagers and cardiologists. The SUS was 71/100 AU, indicating a moderately good usability of the 3D print and visualizations derived from CT and TEE modalities. The separate analysis in surgeons resulted in a SUS of 72/100 AU and thus a similar SUS. This pipeline has wide applicability as mitral valve repairs or replacements are the only treatment option for primary MR recommended by the ESC guidelines, and after GDMT optimization, the primary treatment option for SMR [5]. So, alongside efficient strategies to optimize GDMT, improving interventional efficiency is a way to improve the recommended and proven therapies for all types of MR [5, 19].

Surgical planning using 3D printing has in various applications of abdominal, vascular and orthopedic surgery been shown to improve indicators of effectiveness in surgery such as operating time and bleeding [20, 21, 22, 23]. Recent advancements in 3D printing and the applications of 3D printing workflows in cardiothoracic surgery have demonstrated close to nature flexibility of mitral valve models. In combination with the extensively performed TEE and CT imaging of the heart, these technologies open up the possibility for flexible patient‐specific 3D modeling and simulation in cardiac surgeries and interventions. Previous studies have published pipelines for 3D printing from TEE [24, 25, 26, 27]. Multimodality 3D printing would however be useful for modeling the mitral valve apparatus; as CT can provide superior visualization of the papillary muscles and is not bound to axial restrictions for cardiac chamber imaging, while TEE is superior for visualizing the MV leaflets [27, 28]. This pipeline describes and evaluates such a multimodality pipeline for modeling of the mitral valve apparatus using synergistic information from both modalities. A previous multimodality solution for modeling of the mitral valve apparatus for interventional planning is described by Gosnell et al. used for the modeling of a transcatheter mitral valve intervention [28]. This approach however manually aligns CT with TEE for a fusion case. Mathematical algorithms for fusion of imaging modalities have however in several robotic and clinical applications shown superior fusion accuracies compared to manual alignment strategies [11, 29–31]. Similarly, no manual corrections were needed in this fusion pipeline based on visual inspection of a dedicated imaging cardiologist. Based on these feasibility tests and previously performed alignments tests using mutual information, mutual information based algorithms seems to be an accurate method for the fusion of CT and US modalities for multiple applications [11, 29, 30]. This workflow provides an effective pipeline for multimodality patient‐specific models of the mitral valve to be able to better simulate the underlying anatomy for surgical preparation.

This pipeline was developed by cardiologists and medically trained technicians. Within the development process, close collaboration was maintained to ensure straightforward interpretation of the visualization and facilitate quality control. For the implementations of similar pipelines in the future, we recommend a similar support structure to combine technical know‐how and clinical expertise to enhance pipeline development. Using the feedback from independent specialists, we derived several straightforwardly implementable improvements of the presented pipeline, and improvements that require more development time. A suggestion to separate the valvular leaflets based on temporal information on the TEE should be straightforward and this correction can easily be performed on a 3D print. The suggestion to include a different color for pathological areas is implementable on some commercially available 3D printers. The suggested indication of regurgitation flow directionality would require manual editing and a development process to display regurgitation flow effectively in a 3D‐printed model.

### 4.1 Limitations

This study has limitations that should be taken into account. Multiple software programs are used in this pipeline, which lengthens the time needed to perform a fusion in a clinical implementation. However, considering that the algorithms used during the fusion process are publicly available, this fusion methodology could be implemented within one software program. As this is a feasibility study, the pipeline is tested on retrospective data, and the number of clinical cases tested using the pipeline is limited.

### 4.2 Future Perspectives

As surgeons indicated a moderately good usability of this pipeline with clear and straightforwardly applicable directions for improvements, a more widespread application is warranted to further study pipeline applicability and clinical effectiveness in more cases. To achieve this, the workflow should be applied in a prospective setting where the valvular leaflets should be manually separated in the 3D print. Transferring this pipeline to a prospective setting can, for example, be done in a setting of a (cluster) randomized controlled trial in which the intervention group prepares for a (novel) intervention using a patient‐specific 3D visualizations and 3D prints. Since the TEE and CT imaging are done before any intervention or Heart Team decision, a fusion pipeline can be applied prospectively in the planning phase for any mitral valve intervention. In order to apply this intervention in a more purposeful way, its effectiveness can be tested among surgeons in training or among surgeons practicing for a novel (minimally invasive) surgical procedure. Endpoints of surgical effectiveness should be the eventual goal here to investigate if such a strategy should become the standard for the learning of surgical techniques or in difficult cases. Developments in software programs have resulted in the ability to simulate various cardiac procedures virtually, primarily based on CT imaging (examples include FEOPS, Gent, Belgium; 3Mensio, Pie medical imaging, Maastricht, the Netherlands; or Mimics Planner for Structural Heart Interventions, Mimics, Leuven, Belgium). The presented fusion algorithm can be implemented in such software programs to enhance leaflet visualization in such a virtual simulation or potentially improve simulation by providing information on the leaflet pathology visualized on TEE. Virtual and augmented reality simulations provide a way to virtually simulate a procedure, and this fusion pipeline could also be used in such a simulation to better visualize patient‐specific anatomy. Physical 3D‐printed models do however have the advantage of adding a sense of realism by providing tactile feedback to the operator [32].

## 5 Conclusion

The presented pipeline with digital planning and personalized 3D printing has reached the technology readiness level for application in mitral valve interventions, and larger prospective studies on applicability and clinical endpoints. Small improvements can enhance the usability as assessed by independent clinicians involved in mitral valve interventions who rated the visualizations and 3D prints with a moderately good usability. Comprehensive and efficient interactions between clinicians and technical staff are recommended to establish well‐designed novel technical pipelines for personalized models.

## Conflicts of Interest

Prof. Dr. F. W. Asselbergs received grant funding from the European Union Horizon scheme (AI4HF 101080430 and DataTools4Heart 101057849). Dr. M. J. Schuuring received an independent research grant from AstraZeneca to the research institute and is supported by Stichting Hartcentrum Twente. The remaining authors declare no competing interests.

## Supporting information



Supporting Information

## Data Availability

Anonymized participant data can be made available upon requests directed to the corresponding author. Proposals will be reviewed on the basis of scientific merit, ethical review, available resources, and regulatory requirements. After approval of a proposal, anonymized data will be made available for reuse.
