# Supplementary material for: Development of an Innovative Pipeline With Fusion, Digital Planning, and Three‐Dimensional Printing to Improve Mitral Valve Interventional Care
Source: Echocardiography. 2025 May 20;42(5):e70184. doi: 10.1111/echo.70184 (PMC12090884; doi:10.1111/echo.70184)
Supplement: Supplementary file 1 — Supporting Information [file ECHO-42-e70184-s001.docx]

**Supplementary information**

**Parameter setting**

FixedInternalImagePixelType "float"
MovingInternalImagePixelType "float"
FixedImageDimension 3
MovingImageDimension 3
UseDirectionCosines "true"
Registration "MultiResolutionRegistration"
Interpolator "BSplineInterpolator"
ResampleInterpolator "FinalBSplineInterpolator"
Resampler "DefaultResampler"
FixedImagePyramid "FixedSmoothingImagePyramid"
MovingImagePyramid "MovingSmoothingImagePyramid"
Optimizer "AdaptiveStochasticGradientDescent"
Transform "AffineDTITransform"
Metric "AdvancedMattesMutualInformation"
AutomaticScalesEstimation "true"
AutomaticTransformInitialization "true"
AutomaticTransformInitializationMethod "CenterOfGravity"
HowToCombineTransforms "Compose"
NumberOfHistogramBins 32
ErodeMask "false"
Scales 1.0e+03 1.0e+03 1.0e+03 3.0e+038 3.0e+038 3.0e+038 3.0e+038 3.0e+038 3.0e+038 -1 -1 -1
NumberOfResolutions 2
MaximumNumberOfIterations 3000
NumberOfSpatialSamples 10000
CheckNumberOfSamples "false"
NewSamplesEveryIteration "true"
ImageSampler "RandomCoordinate"
BSplineInterpolationOrder 1
FinalBSplineInterpolationOrder 1
DefaultPixelValue 0
WriteResultImage "true"
ResultImagePixelType "float"
ResultImageFormat "nii"
MaximumNumberOfSamplingAttempts 100
